# Performance of growing lambs supplemented with ground licuri (*Syagrus coronata*)

**DOI:** 10.5713/ajas.20.0199

**Published:** 2020-08-24

**Authors:** Adin Daza, Jocely G. Souza, Joao Paulo I. S. Monnerat, Claudio V. D. M. Ribeiro

**Affiliations:** 1 Department of Veterinary Medicine and Animal Science, Federal University of Bahia, Salvador, 40.170-110, Brazil; 2 Department of Animal Science, Federal University Rural of Pernambuco, Recife, 50.670-901, Brazil

**Keywords:** Alternative Feed, Digestibility, Intake, Physically Effective Fiber, *Syagrus coronata*

## Abstract

**Objective:**

The aim of this study was to determine the effect of dietary ground licuri on lamb performance.

**Methods:**

Forty male lambs were used in a completely randomized design to test the effects of 0, 5, 10, and 15 g/kg of ground licuri added to diets. The trial lasted for 75 days. Intake, digestibility, physically effective neutral detergent fiber, and chewing activity were estimated. Blood samples were taken on day 45 to determine the concentrations of glucose, urea, non-esterified fatty acids, and triglycerides. Average daily gain (ADG) were determined on the last day of the experimental trial.

**Results:**

Licuri inclusion markedly increased dietary neutral detergent fiber and ether extract (EE) content, but it decreased dry matter (DM) intake. However, the intake and digestibility of EE linearly increased. The ADG decreased linearly (p<0.05) with licuri inclusion. Licuri had no effect (p>0.05) on the concentrations of blood metabolites; however, blood urea increased (p<0.05), while serum glucose decreased (p<0.05).

**Conclusion:**

The physically effective fiber of ground licuri is similar to Tyfton hay and licuri inclusion decreases lamb performance due to a decreased in DM intake.

## INTRODUCTION

Corn and soybena meal are feed ingredients widely used in animal diets and both represent a large proportion of the cost of feeding. Therefore, the evaluation of lower cost ingredient alternatives to replace traditional feeds is of great importance in maintaining or improving the profitability of sheep production.

The licuri ( *Syagrus coronata*) is a palm tree found at sheep farms in semiarid regions. Its fruits (or coconuts) are drupes with abundant oily endosperm and a fibrous mesocarp. It is an important food resource for humans and animals in the northeast Brazil, although licuri is mainly fed as licuri cake in animal nutrition, which is used to increase the concentration of ether extract (EE) and neutral detergent fiber (NDF) in the diet [1,2]. The inclusion of 24% licuri cake in diets fed to lambs has led to a 39% reduction in intake and a decreased weight gain (WG) and average daily gain (ADG) [2]. On the other hand, a 17% inclusion of licuri has been reported to improve feed conversion and to increase ADG [3], and no changes in the dry matter intake (DMI) of lambs was observed with 16% inclusion of licuri cake [1]. In addition, the fatty acid profile of licuri oil is different from traditional oil sources; it is highly saturated, with high percentages of medium chain fatty acids (MCFA), mainly lauric and myristic acids [4]. Fat is commonly used to increase the energy density in animal diets; thus, licuri may be used for this purpose. Improved sensory attributes of meat from lambs fed licuri cake has been reported, with a concomitant heathier meat fatty acid profile [5].

Ground licuri fruit is widely used in extensive and semi- extensive systems where the plant is abundant. However, to the best of our knowledge, no scientific studies have been done with licuri fruit inclusion in lamb diets and how it affects animal performance. The licuri fruit (seed plus endocarp) is a non-forage fiber source (NFFS) with a peculiar fatty acid profile that could behave similarly to licuri cake. Therefore, the aim of this study was to test the hypothesis that low levels (up to 15%) of ground licuri fruit could be added to lamb diets without impact animal performance.

## MATERIALS AND METHODS

### Protocol, animals, and general procedures

This study was conducted in conformity with the Brazilian legislation on experimentation involving the use of animals by the National Council of Experimental Control (CONCEA) and approved by the Ethics Committee in Animal Use (CEUA) of the Federal University of Bahia (protocol number 22/2011).

Forty, non-castrated, six-month-old crossbred Dorper× Santa Ines lambs, weighing an average of 20.89 (±3.97) kg of body weight (BW), were used in a completely randomized design, with 10 replicates per treatment. The experiment lasted 75 days plus 10 days of adaptation. A dose of 1 mL per animal of ivermectin (Baymec DE, Bayer, SP, Brazil) was used to control external and internal parasites. The lambs were housed in individual pens (1.00×1.00 m).

### Ingredients, diets, and chemical composition

The Tifton hay was bought from a commercial farm and shredded with a grass chopper machine (model TF150, Laboremus Ltd, Campina Grande, Brazil) with a 2-cm screen. The sisal by-product was acquired from the Companhia Sisal do Brasil (COSIBRA) farm. All diets were composed of Tifton hay and sisal silage (2:1 ratio) as forage sources; the concentrate was based on corn and soybean meal (Table 1).

The licuri fruits were harvested and ground with a grass chopper machine (TFC150 MIN500, Incomagri, Itapira, Brazil) with a 2-mm sieve before mixing with the concentrate. The treatments (Table 2) consisted of 4 levels of ground licuri in the diets (dry matter [DM] basis), as follows: i) control (no licuri), ii) 5 g/kg, iii) 10 g/kg, and iv) 15 g/kg of ground licuri in the total diet DM.

The non-fiber carbohydrates (NFC) were calculated ac cording Hall [6], total digestible nutrient (TDN) content was estimated as follows: TDN (g/kg) = (TDN intake/DMI)×100, where TDN intake was calculated using the following equation [7]: TDN (g/d) = (CP_i_ − CP_f_)+(2.25×[EE_i_ − EE_f_])+(TC_i_ − TC_f_), where CP, crude protein; EE, ether extract; and TC, total carbohydrate; and i and f represent intake and feces, respectively.

### Intake and digestibility

The lambs were fed twice daily at 8:00 h and 16:00 h, ensuring a 10% orts and water *ad libitum*. Individual intake was recorded daily, and DMI was estimated (offered minus orts).

The digestibility trial was performed from day 41 to 45 of confinement. Orts and feces were collected from 20 lambs by the total collection method, in which the feces were collected individually using fecal collection bags. In addition, total mixed ration samples were collected during the same period. The samples were mixed, and a composite sample was taken, dried at 55°C, and stored for further analysis.

All samples of ingredients, feed, feces, and orts were ground in a Wiley mill (Wiley mill TE-680, TECNAL, Piracicaba, Brazil) with a 1-mm screen and analyzed according to the AOAC [8] for DM (method 934.01), ash (method 942.05), nitrogen (method 2001.11), and EE (method 920.39). The NDF and ADF followed a procedure using heat stable amylase [9]. The digestibility coefficients (DC) of DM, organic matter (OM), CP, EE, NDF, NFC, and TDN were calculated using the following equation: DC = (nutrient intake [g] − nutrient excretion [g])/nutrient intake (g).

### Physically effective neutral detergent fiber and chewing activity

On days 66 and 67 of the study, approximately 150 to 250 g of total mixed ration were collected for the determination of particle size. Sample particle distributions were determined using the Penn State Particle Separator [10]. The physically effective factor (pef) values were determined as the total proportion of DM retained on 2 sieves: 19.0 and 8.0 mm [10] (pef_8.0_) or on 3 sieves: 19.0, 8.0, and 1.18 mm [11] (pef_1.18_).

The physically effective NDF (peNDF) values were calcu lated by multiplying pef_8.0_ and pef_1.18_ by the NDF content of the samples (DM basis) to obtain peNDF_8.0_ and peNDF_1.18_, respectively [12]. Then, the dietary intake of pef_8.0_, pef_1.18_, peNDF_8.0_, and peNDF_1.18_ were estimated (Table 4). Ruminanting and chewing activity were continuously monitored and recorded every 5 min for 24 h on day 67 of the experimental period. Activity was recorded as eating, ruminating or idle, and each activity was assumed to persist for the entire 5-min interval. The data were used to estimate the ruminating time and ruminating per gram of NDF [13]. Total time spent chewing was calculated as the total time spent eating and ruminating.

### Blood parameters

Blood samples were taken from the external jugular veins using beveled needles and 10-mL tubes containing ethylenediaminetetraacetic acid. The tubes were centrifuged at 800 *g* for 15 min, and the serum samples were placed in sterile test tubes and stored at −20°C until further analysis. Blood samples were collected at 6 h after feeding on day 45 of the experimental period. Blood triglycerides (TG; enzymatic-Thinder method-Labtest, ref. 87; Lagoa Santa, Brazil), urea (urease endpoint method-Labtest, ref. 27), glucose (kinetic method-Labtest, ref 133), albumin (Bromocresol Green method-Labtest, ref. 19), creatinine (colorimetric method, endpoint Kit-Labtest, ref 35), non-esterified fatty acids (NEFA; colorimetric method-RANDOX; Crumlin, Ireland), aspartate aminotransferase (AST; kinetic method UV – Doles, Goiania, Brazil), and alanine aminotransferase (ALT; kinetic method UV - Doles) were analyzed using spectrophotometer (Bio-Rad Laboratories, Hercules, CA, USA) at the wavelength specified by each procedure.

### Animal performance

On days 0 and 75 of the experimental period, the animals were fasted for 16 h and weighed to determine BW, average BW, WG, ADG, and BW at slaughter (final BW). The feed conversion ratio (FCR; grams feed per grams WG) was calculated for each individual lamb.

### Statistical analysis

The data were analyzed using PROC MIXED of SAS (SAS Inst. Inc., Cary, NC, USA version 9.4) in a completely randomized design. Heterogeneity of variance was tested by the command REPEATED and used when significant. Polynomial contrasts were used to test the linear and quadratic effects of licuri supplementation on all parameters. Initial BW and blood parameters sampled before the experimental period were tested as covariables and used when significant. Significance was declared at p≤0.05.

## RESULTS

### Intake and digestibility

Licuri inclusion showed a negative linear effect (p≤0.05) on DMI. The total DCs of DM, OM, CP, and NDF were not affected by the treatments (Table 3). In contrast, the inclusion of licuri increased (p≤0.05) EE but decreased NFC and TDN digestibility.

### Physically effective neutral detergent fiber and chewing activity

Licuri increased (p≤0.05) intakes of pef_8.0_, pef_1.18_, and peNDF_8.0_ (Table 4). However, the proportion of particles retained on the pan decreased (p≤0.05), the particles retained on the 1.18-mm sieves did not change, and their peNDF_1.18_ values had a quadratic response. Both, ruminating and total chewing time, linearly increased (p≤0.05) with dietary licuri inclusion (Table 5). Only total chewing time per gram of peNDF_8.0_ did not change.

### Blood parameters

Inclusion of licuri in the diets did not affect daily averages of blood serum creatinine, albumin, TG, NEFA, ALT, or AST concentrations; however, serum urea concentration increased, and glucose concentration decreased (p≤0.05; Table 6).

### Performance

Inclusion of ground licuri in the diets decreased animal performance (p≤0.05); however, the inclusion of licuri had a quadratic effect on feed conversion (Table 7).

## DISCUSSION

In ruminant diets, NFFS have been added as a replacement for either concentrate [14,15] or forages [14]. The results were depended on the level of inclusion, ruminal digestibility of the NDF fraction, and the magnitude of the fiber effectiveness of the NFFS, among others. Because licuri cake is similar in NDF content to licuri fruit, this study followed the same nutritional management, in which licuri was supplemented in the concentrate.

Voluntary DMI and ruminal filling has been found to be related to bulk density of forages [16], which is negatively correlated to intake and animal performance. The DMI reduction suggests a ruminal filling effect, probably caused by the 28.4% increase in dietary NDF between the control diet and the diet with 15% licuri. The NFFS have fiber that ferments and passes rapidly from the rumen, which would not decrease intake; therefore, an increase in dietary NDF to stimulate chewing and saliva secretion may be an important factor with diets containing NFFS. In the present study, licuri increased dietary peNDF which resulted in a decrease in DMI (Table 3). Although the extent and potential of licuri NDF ruminal digestibility has not been determined, we speculate that its ruminal fiber digestibility may be lower than other traditional NFFS.

There are other factors (fatty acid profile, palatability etc.) that may have played a role in the lower intake associated with dietary licuri. High concentrations of MCFAs have been reported for licuri [4]. The MCFA have physical properties (lower melting point) similar to unsaturated fatty acids, which increase cellular membrane fluidity. An increase in MCFA has been associated with a decrease in DMI [17]. Also, licuri oil has exhibited bactericide and bacteriostatic effects [17]. Therefore, the rationale regarding the effect of unsaturated fatty acids impairing ruminal fiber digestion and the filling effect may extend to MCFA as well. The MCFA are also absorbed by the abomasum, reaching the liver by the portal hepatic system. These fatty acids have a high propensity for oxidation, behaving in a similar way to glucose. Because metabolic fuels reaching the liver may increase satiety [16], licuri supplementation increased intake and absorption of MCFA-rich EE, which may have decreased DMI by a post-absorptive satiety mechanism as well. The lack of treatment effect on DM digestibility was associated with the opposite responses on the digestibility of NFC and EE.

Licuri presented 91 g/kg of EE (DM basis), and this may explain the 92% increase in EE intake between the control and the treatment with 15% licuri inclusion. The increase in EE intake has been found to increase its digestibility as a function of the dilution of the endogenous EE and by the intestinal digestibility of fatty acids that are inversely related their melting point [18]. Increasing the proportion of MCFAs in the dietary EE may also increase total EE digestibility. Because dietary corn decreased with licuri inclusion (Table 2), NFC digestibility decreased. Therefore, the lower contribution of corn starch was responsible for the reduction in NFC digestibility.

The peNDF is related to DMI, particle size, particle shape, fragility, moisture, type of preservation, and ratio of eating time to ruminating time [12]. The physical and chemical nature of dietary NDF largely drives chewing activity. Licuri fruit has low *in vitro* NDF digestibility (26.77%) and a high lignin content (22.69%) [19], values lower than Tifton hay, which has *in vitro* DM digestibility of 58.7% with a lignin content of 4.8% [20].

The total chewing time is strongly influenced by the forage content and feed size particles. Total chewing and ruminating time increases concomitantly with dietary peNDF [21–23]. As the proportion of NDF in the diet increased, each unit of DM was chewed for a longer period of time, mainly during eating, which resulted in a loss of ruminating time per unit of NDF consumed [24]. Ruminating and chewing were also expected to increase with licuri supplementation. The lack of effect on ruminating and total chewing times can be explained by the reduced intake with licuri supplementation. Increasing forage particle length increased peNDF intake by goats, but linearly decreased DMI [23]. The effect of NFFS on DMI may differ whether they are substituted for forage or concentrate; they have been associated with a decrease in DMI when NFFS replaced concentrate [16].

Licuri inclusion resulted in increased ruminating and total chewing times per gram of DM and NDF intake, as well as ruminating and chewing times per gram of both peNDF (Table 5). Rumination time and chews per unit of consumed fiber are considered to be indices of fragility or the susceptibility of the cell wall to break down [24]. The majority of NFFSs have been found to decrease rumination time (min/d); therefore, the forage fiber requires adjustments to maintain chewing activity [25], with few exceptions which possess greater physical effectiveness [26].

The lower performance observed as licuri was added to treatment diets was a consequence of the linear decrease in DMI; there was a 25.1% reduction between the lowest (control) and the highest level of licuri inclusion. The decrease in DMI led to a lower nutrient intake, which negatively affected the performance and fat thickness (FT) of the lambs. In fact, the ADG observed was 171 g/d, a value slightly higher than the value (160 g/d) observed in Santa Ines×Dorper lambs fed licuri cake [2]. However, the FT was lower in our treatments, ranging from 1.41 to 0.81 mm. On the other hand, Santos et al [1] reported a quadratic effect (p<0.05) on animal performance without changes in the FT of longissimus muscles when lambs were fed licuri cake.

The decrease in ADG may be due to the lowest intake of CP and TDNs, as ground licuri was included in the diets, which may have resulted in a decreased muscle accretion and lower deposition of subcutaneous fat. Control lambs had the highest WG with similar FCR. The proportional decrease in both DMI and ADG caused a similar FCR among treatment diets, averaging 4.69.

Blood creatinine is a product of nitrogen metabolism; it is considered an index of endogenous protein catabolism, and it is also used to asses renal function [27]. The albumin can reflect protein deficiency. Despite the reduction in DMI and ADG, supplementing licuri cake had no effect on serum albumin or creatinine [28].

Serum urea showed an increasing linear effect among treatments, although the diets were isonitrogenous. Higher amounts of rumen dietary carbohydrate have been associated with increased sequestration of NH_3_-N into microbial proteins [29]. When licuri or its by-products are added to diets, part of the energy from corn starch is substituted by the energy from licuri fat, which diminishes the amount of energy that is ruminally degraded. In addition, the lower NFC digestibility may have decreased the sequestration of urea and increased blood volume as dietary licuri levels increased. The same response has been observed in lambs fed licuri cake [5]. The decrease in the concentration of serum glucose was caused by the proportional reduction of dietary NFC as licuri was added to diets. Lower dietary starch and/or lower ruminal NFC digestibility would decrease liver propionate gluconeogenesis [16].

The blood parameters in this study were kept within nor mal reference values [30], except for the serum concentration of TG, which was higher in all treatments. Feeding licuri cake to lambs has been shown to increase blood TGs, with no effect on the concentration of AST and ALT [28]. The quadratic response of ALT concentration, with the lowest value on the 5% licuri treatment, should be investigated further; however, we speculated that it happened by chance only.

Ground licuri is an NFFS that decreases feed intake. Because licuri has high NDF and MCFA concentration it negatively impacts nutrient intake and performance concomitantly. The licuri peNDF_1.18_ is similar to that of Tifton hay and, thus, has the potential to be used as a NFFS. Therefore, future studies should determine the optimum level of inclusion of ground licuri as a replacement for forage in high concentrate diets for ruminants.

## Figures and Tables

**Table 1:** Nutrient composition, particle distribution, physical effectiveness factor (pef), and physically effective neutral detergent fiber (peNDF) of dietary ingredients

Item	Ingredients				

	Tifton hay	Sisal silage by-products	Ground corn	Soybean meal	Ground licuri
Nutrients1)					
DM (g/kg fresh weight)	907	120	872	874	914
Organic matter	939	908	989	936	976
Mineral matter	61	92	11	64	24
Crude protein	62	67	97	518	60
Ether extract	11	13	31	26	91
Neutral detergent fiber	738	274	113	177	672
Acid detergent fiber	350	188	22	59	393
Cellulose	249	176	29	66	188
Hemicellulose	316	110	56	34	237
Lignin	44	93	22	22	165
Non-fibrous carbohydrates	128	554	766	226	155
Particle size distribution2)					
19 mm	67.6	5.94	0.00	0.00	0.00
8 mm	11.7	9.54	0.00	0.00	29.2
1.18 mm	13.7	65.5	54.3	56.1	62.0
Pan	7.02	19.0	45.7	43.9	8.80
pef_8.0_	0.79	0.15	0.00	0.00	0.29
pef_1.18_	0.93	0.81	0.54	0.56	0.91
peNDF_8.0_ (% of DM)	58.3	4.11	0.00	0.00	19.5
peNDF_1.18_ (% of DM)	68.6	22.2	6.11	9.93	61.2

DM, dry matter.

1) Expressed as g/kg DM.

2) Particle size distribution of ingredients was measured using the Penn State Particle Separator (Kononoff et al [11]); pef_8.0_ and pef_1.18_ = physical effectiveness factor determined as the proportion of particles retained on 2 sieves (Lammers et al [10]) and on 3 sieves (Kononoff et al [11]), respectively; peNDF_8.0_ and peNDF_1.18_ = physically effective NDF determined as NDF content of the diet multiplied by pef_8.0_ and pef_1.18_, respectively.

**Table 2:** Ingredients, nutrient composition, particle distribution, physical effectiveness factor (pef), and physically effective neutral detergent fiber (peNDF) contents of experimental diets

Item	Treatments1)			

	0	5	10	15
Ingredients2)				
Ground Licuri	0	56	112	169
Vitamin/mineral mix	11	11	11	11
Soybean meal	142	145	146	149
Ground corn	376	321	263	208
Urea/*ammonium sulphate*	11	11	11	11
Tifton hay	290	290	289	288
Sisal silage by-products	167	167	167	165
Nutrients2)				
DM (g/kg fresh weight)	757	762	764	768
Organic matter	942	942	941	940
Mineral matter	58	58	59	60
Crude protein	168	168	166	165
Ether extract	21	24	28	31
Neutral detergent fiber	328	359	390	421
Acid detergent fiber	150	170	191	211
Cellulose	122	131	140	148
Hemicellulose	136	146	156	166
Lignin	40	48	56	64
Non-fibrous carbohydrates	451	417	382	347
Particle size distribution3)				
% of DM retained on sieves				
19.0-mm	20.9	20.9	20.8	20.7
8.0-mm	4.95	6.32	7.66	8.99
1.18-mm	43.1	43.81	44.4	45.0
Pan	31.0	28.9	27.1	23.1
pef_8.0_	0.26	0.27	0.28	0.30
pef_1.18_	0.69	0.71	0.73	0.75
peNDF_8.0_ (% of DM)	8.50	9.79	11.1	12.5
peNDF_1.18_ (% of DM)	22.7	25.5	28.5	31.5

DM, dry matter.

1) Percentage inclusion of ground licuri in the diets.

2) Expressed as g/kg DM.

3) Particle size distribution of mixed diets was measured using the Penn State Particle Separator (Kononoff et al [11]); pef_8.0_ and pef_1.18_ = physical effectiveness factor determined as the proportion of particles retained on 2 sieves (Lammers et al [10]) and on 3 sieves (Kononoff et al [11]), respectively; peNDF_8.0_ and peNDF_1.18_ = physically effective NDF determined as NDF content of the diet multiplied by pef_8.0_ and pef_1.18_, respectively.

**Table 3:** Least square means of nutrients intake and digestibility of lambs fed increasing levels of ground licuri

Item	Treatments1)				SEM	p-value2)	
	
	0	5	10	15		L	Q
Intake (g/d)							
Dry matter	936	851	864	700	39.01	<0.01	0.32
Organic matter	872	803	807	650	36.31	<0.01	0.24
Crude protein	135	139	138	113	5.83	<0.01	0.01
Ether extract	13	14	27	25	0.84	<0.01	0.19
Neutral detergent fiber	186	176	158	126	8.30	<0.01	0.20
Non-fibrous carbohydrates	313	283	287	208	11.96	<0.01	0.05
Total digestible nutrients	895	825	846	684	37.65	<0.01	0.23
Digestibility coefficients							
Dry matter	0.654	0.661	0.646	0.614	0.002	0.10	0.30
Organic matter	0.686	0.691	0.675	0.643	0.002	0.06	0.27
Crude protein	0.729	0.739	0.755	0.764	0.002	0.13	0.98
Ether extract	0.743	0.816	0.874	0.836	0.002	<0.01	<0.01
Neutral detergent fiber	0.529	0.548	0.547	0.491	0.002	0.27	0.11
Non-fibrous carbohydrates	0.858	0.849	0.815	0.802	0.001	<0.01	0.83
Total digestible nutrients	0.795	0.789	0.767	0.747	0.001	0.02	0.62

SEM, standard error of the mean.

1) Percentage inclusion of ground licuri.

2) Probabilities of orthogonal contrasts for testing linear (L) and quadratic (Q) effects.

**Table 4:** Least square means of particle size distribution, physical effectiveness factor (pef), and physically effective neutral detergent fiber (peNDF) contents in diets consumed by lambs fed increasing levels of ground licuri

Item1)	Treatments2)				SEM	p-value3)	
	
	0	5	10	15		L	Q
Diets consumed (adjusted for particle size of orts)							
% DM retained on sieve							
19.0-mm	20.3	20.7	21.7	23.3	0.30	<0.01	0.07
8.0-mm	3.37	3.72	5.37	6.20	0.40	<0.01	0.56
1.18-mm	44.2	45.4	44.0	44.5	0.43	0.76	0.40
Pan	32.1	30.9	28.8	26.3	0.23	<0.01	<0.01
pef_8.0_	0.24	0.24	0.27	0.29	0.01	<0.01	0.13
pef_1.18_	0.68	0.69	0.71	0.74	0.01	<0.01	0.01
peNDF_8.0_ (% of DM)	9.09	9.22	9.19	11.0	0.26	<0.01	<0.01
peNDF_1.18_ (% of DM)	26.0	26.4	24.1	26.7	0.40	0.92	<0.01

SEM, standard error of the mean; DM, dry matter.

1) Particle size distribution of mixed diets or orts was measured using the Penn State Particle Separator (Kononoff et al [11]); pef_8.0_ and pef_1.18_ = physical effectiveness factor determined as the proportion of particles retained on 2 sieves (Lammers et al [10]) and on 3 sieves (Kononoff et al [11]), respectively; peNDF_8.0_ and peNDF_1.18_ = physically effective NDF determined as NDF content of diets multiplied by pef_8.0_ and pef_1.18_, respectively.

2) Percentage inclusion of ground licuri.

3) Probabilities of orthogonal contrasts for testing linear (L) and quadratic (Q) effects.

**Table 5:** Least square means of ruminating and chewing activity of lambs fed increasing levels of ground licuri

Item1)	Tratements2)				SEM	p-value3)	
	
	0	5	10	15		L	Q
Ruminating							
Min/g of DM	0.41	0.49	0.46	0.63	0.03	<0.01	0.21
Min/g of NDF	1.10	1.32	1.40	1.62	0.08	<0.01	0.98
Min/g of peNDF_8.0_	4.49	5.44	4.99	5.89	0.37	0.03	0.94
Min/g of peNDF_1.18_	1.59	1.89	1.90	2.31	0.14	<0.01	0.71
Chewing							
Min/g of DM	0.66	0.72	0.69	1.02	0.06	<0.01	0.02
Min/g of NDF	1.78	1.92	2.11	2.76	0.14	<0.01	0.07
Min/g of peNDF_8.0_	7.43	8.00	7.82	9.02	0.55	0.07	0.58
Min/g of peNDF_1.18_	2.63	2.78	2.97	3.55	0.21	<0.01	0.33

SEM, standard error of the mean; DM, dry matter; NDF, neutral detergent fiber; peNDF, physically effective NDF.

1) peNDF_8.0_ and peNDF_1.18_ = physically effective NDF determined as NDF content of mixed diet multiplied by pef_8.0_ and pef_1.18_, respectively; pef_8.0_ and pef_1.18_ = physical effectiveness factor determined as the proportion of particles retained on 2 sieves (Lammers et al [10]) and on 3 sieves (Kononoff et al [11]), respectively.

2) Percentage inclusion of ground licuri.

3) Probabilities of orthogonal contrasts for testing linear (L) and quadratic (Q) effects.

**Table 6:** Least square means of blood parameters of lambs fed increasing levels of ground licuri

Items	Treatments1)				SEM	p-value2)	
	
	0	5	10	15		L	Q
Creatinine (mg/dL)	1.06	1.05	1.04	1.08	0.08	0.89	0.75
Albumin (g/dL)	3.84	3.84	3.73	3.74	0.09	0.33	0.97
Urea (mg/dL)	42.03	43.76	46.86	48.29	1.50	0.01	0.29
Glucose (mg/dL)	99.9	94.3	97.8	93.6	1.50	0.03	0.66
NEFA (mmol/L)	0.94	0.94	0.94	0.87	0.04	0.27	0.43
Triglyceride (mg/dL)	108	106	110	101	2.83	0.17	0.18
ALT (UI/L)	20.5	14.4	18.8	18.6	2.50	0.91	0.25
AST (UI/L)	100	97.2	101.8	69.7	9.84	0.55	0.65

SEM, standard error of the mean; NEFA, non-esterified fatty acids; ALT, alanine aminotransferase; AST, aspartate aminotransferase.

1) Percentage inclusion of ground licuri seed.

2) Probabilities of orthogonal contrasts for testing linear (L) and quadratic (Q) effects.

**Table 7:** Least square means of performance parameters of lambs fed increasing levels of ground licuri

Item	Treatments1)				SEM	p-value2)	
	
	0	5	10	15		L	Q
Initial BW (kg)	20.6	21.1	21.6	20.2	1.29	0.897	0.470
Final BW (kg)	36.7	33.5	34.8	32.3	0.77	<0.01	0.62
WG (kg)	15.5	12.3	13.6	11.1	0.74	<0.01	0.57
ADG (g/d)	215	170	188	154	10.4	<0.01	0.58
Fat thickness (mm)	1.19	1.41	1.17	0.81	0.12	0.02	0.03
FCR	4.44	5.04	4.68	4.61	0.16	0.83	0.05

SEM, standard error of the mean; BW, body weight; WG, weight gain; ADG, average daily gain; FCR, feed conversion ratio.

1) Percentage inclusion of ground licuri seed.

2) Probabilities of orthogonal contrasts for testing linear (L) and quadratic (Q) effects.
